# Introducing chirality in porous organic cages through solid-state interactions†

**DOI:** 10.1039/d4sc04430d

**Published:** 2024-09-18

**Authors:** Emma H. Wolpert, Kim E. Jelfs

**Affiliations:** a Department of Chemistry, Imperial College London, Molecular Sciences Research Hub, White City Campus Wood Lane London W12 0BZ UK e.wolpert@imperial.ac.uk k.jelfs@imperial.ac.uk +44 20759 43438; b Department of Materials, Imperial College London London SW7 2AZ UK; c I-X Centre for AI in Science, Imperial College London White City Campus W12 0BZ UK

## Abstract

Molecular cages contain an internal cavity designed to encapsulate other molecules, resulting in applications in molecular separation, gas storage, and catalysis. Introducing chirality in cage molecules can improve the selective separation of chiral molecules and add new functionalities due to the realisation of chiral photophysical properties. It has recently been shown that solid-state supramolecular interactions between achiral cages can result in the formation of chiral cavities. Here, we develop a computational technique to predict when achiral cages form chiral cavities in the solid-state through the combination of atomistic calculations and coarse-grained modelling to predict the crystalline phase behaviour. Our focus is on the achiral cage B11, which contains rotatable arene rings on the vertices of the cage that can form propeller-like orientations, inducing a chiral cavity. We show that by using dimer pair calculations, we can inform coarse-grained models to predict the packing of the cage. Our results reveal how the supramolecular interactions drive chirality in the achiral cages without the need for a chiral guest. These findings are a first step towards understanding how we can design chirality through supramolecular interactions by using abstract coarse-grained models to inform design principles for targeted solid-state phase behaviour.

## Abstract 2

Combining dimer pair calculations and coarse-grained modelling to predict the solid-state phase behaviour of porous organic cages and build design principles for introducing chirality into achiral cages.

## Graphical abstract

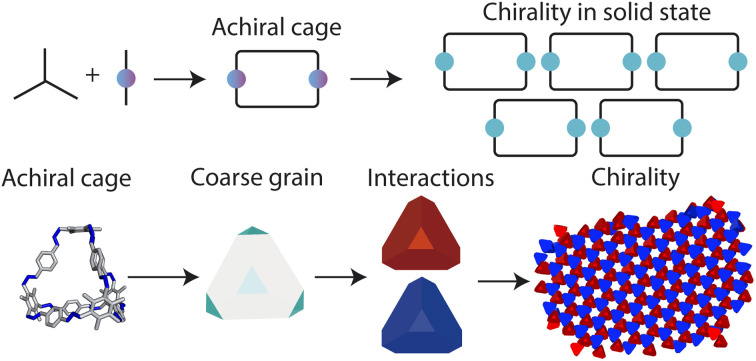

## 1 Introduction

**Fig. 1:**
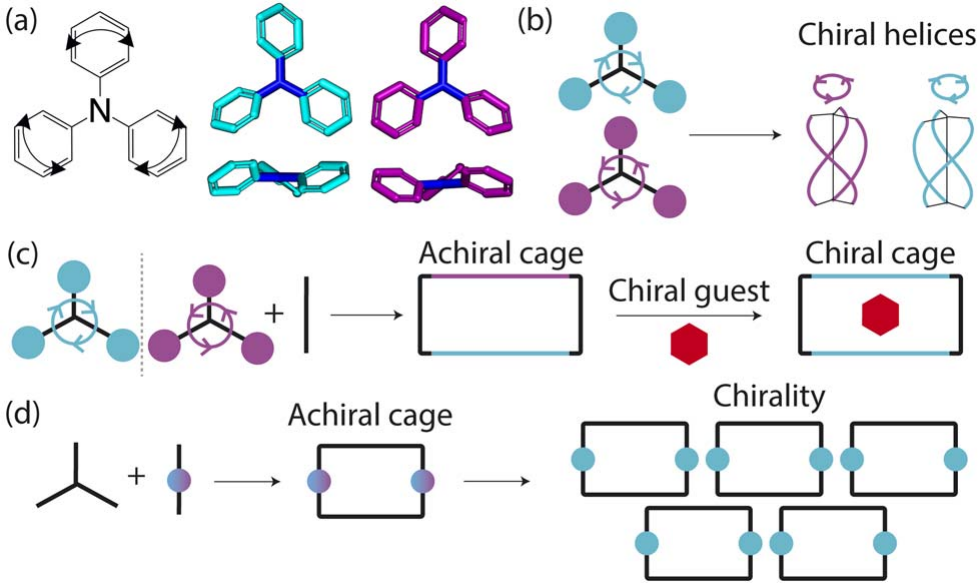
Examples of propeller-like chirality due to supramolecular interactions. (a) Molecules such as triphenylamine (TPA) have propeller-like chirality due to the coupling of the dynamic rotating units such as aromatic rings. This leads to *P*/*M* chiral isomers with clockwise (cyan) and anticlockwise (purple) rotations of the aromatic rings respectively. (b) These tri-aryl type molecules self-assemble to form supramolecular helices with different handedness. (c) Cages formed from precursors with propeller-like chirality are often achiral as the chirality of each component can fluctuate. On addition of a chiral guest (red), the cage becomes chiral as the supramolecular interactions lead to through-space chirality transfer between the components. (d) Achiral cages that do not have chirality in the precursors can have chirality induced into them through supramolecular interactions, resulting in different isomers of the cage molecule on crystallisation leading to helical or propeller-like chirality.

Porous organic cages (POCs) are discrete molecules which contain a permanent internal cavity, providing a wide variety of potential applications such as encapsulation,^1^ molecular separations,^2^ and sensing.^3^ Introducing chirality into the cages adds another level of functionality, as chiral hosts can allow selective recognition and separation of rare gases and chiral molecules^4–8^ and hydrocarbon derivatives,^9,10^ as well as the realisation of photophysical properties such as circular polarised luminescence.^11,12^ Chirality in cages can occur on formation, either through chiral or achiral precursors,^13,14^ or through the addition of a chiral guest inducing chirality into a previously achiral cage. One route to introduce chirality into an achiral cage occurs when the cage comprises of dynamic rotational units (such as aromatic rings) that, on the addition of a chiral guest, cause homodirectional rotation of the dynamic units to bind with the guest (Fig. 1(a and c)).^15^ Without the chiral guest, the rotational units are too far away and so their torsional behaviour is decoupled, leading to achiral cages. The addition of the chiral guest incites the rotational units to have homodirectional rotation as there is a through-space chirality transfer due to the supramolecular interactions between the chiral guest and achiral host.

**Fig. 2:**
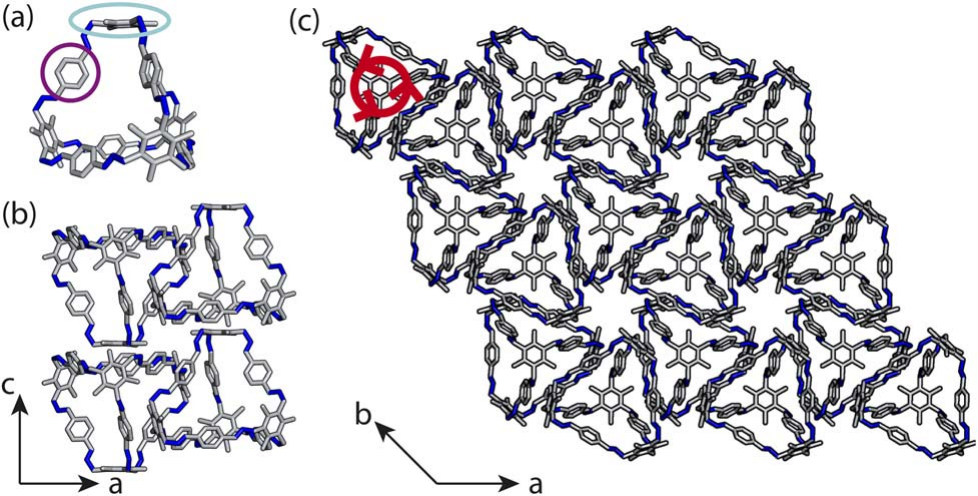
(a) The psuedo-truncated tetrahedral cage B11. This cage contains arene groups on the vertices such that there are three arenes, one of which is circled in purple, around each arene facet of the cage, one of which is circled in cyan. B11 forms a desolvated crystal structure with columns of cages stacking window-to-arene down the *c*-axis, as evident when looking at the *ac* plane in (b). (c) The *ab*-plane of the experimentally reported crystal structure. The red arrows show the propeller like conformations of the arene groups along the vertices around the central axis through each cage's centre.

For non-cage molecules with rotational units such as triphenylamine (TPA) derivatives, as studied by Kim *et al.*,^16^ the rotations of the aromatic rings are coupled, resulting in unidirectional tilting of the rotational units and propeller-like chirality of the molecule alone (Fig. 1(a)). These TPA derivative molecules therefore have two isomers with (*P*)- or (*M*)-propeller chirality that aggregate to form supramolecular helices with one chirality (Fig. 1(b)). This use of supramolecular interactions in the self-assembly of the molecules is able to “lock in” the chirality of the molecules. Similarly, symmetric, achiral cages have been known to crystallise with asymmetric, chiral cavities.^17,18^ Here, the cages can adopt multiple different conformers, with density functional theory (DFT) calculations suggesting that the most stable conformer is achiral. However, on crystallisation the supramolecular interactions direct towards the preferential formation of a chiral conformer, inducing chirality into the previously achiral cage (Fig. 1(d)).^18^ Many molecular cages contain dynamic units within their structure, as the shape persistence of the molecule leading to the open cage structure is often a result of the aromatic rings within the precursors. For example, Greenaway *et al.* combined triamines with a variety of dialdehydes and discovered 25 cages with aromatic rings along the cage vertices that can freely rotate.^19^ These cages often have the arenes along the vertices arranged with three-fold symmetry around the facets of the polyhedral like cages (Fig. 2(a)). Therefore, although the cages are typically achiral, it may be possible for supramolecular interactions upon crystallisation to induce propeller-like chirality into achiral cages.

Here, we test the ability for supramolecular interactions in the solid state to direct chirality on the molecular level in molecular cages. We look at the POC B11 first reported by Greenaway *et al.* in 2018.^19^B11 is formed from the reaction of six (2,4,6-trimethylbenzene-1,3,5-triyl)trimethanamines and four terephthalaldehydes to form a [4 + 6] topology cage that is geometrically the shape of a truncated tetrahedra (Fig. 2(a)). Our primary interest in B11 here is due to its three arene groups arranged on the vertices around the trisubstituted benzene facet (Fig. 2(a)). Unlike in similar cycloimine cages where the helicity, or axial chirality, is an intrinsic property of the cage,^14^B11 does not inherently possess chirality. However, as with other triaryl-type molecules,^16^ it is able to form propeller-like conformations depending on the orientation of the arene groups on the vertices. DFT calculations performed on the single molecule show there is no unidirectional tilting of the aromatic rings on the vertices forming propeller-like chirality,^19^ but these propeller-like conformations could potentially be accessible through favourable supramolecular interactions, introducing chirality in the otherwise achiral cages. From examining the solid-state structure of B11 as explored by Greenaway *et al.*,^19^ propeller like conformations form around the *c*-axis in the *ab*-plane of the crystal structure (Fig. 2(c)). As the propeller-like conformation is not seen in the single molecule (based on the reported DFT calculations), we sought to determine here whether the emergence of the propeller-like behaviour in the solid state is an artifact of the dynamic nature of the aryl groups, or whether the solid-state supramolecular interactions between the cages drive the chirality.

Recently, we developed a new methodology to predict the packing of POCs based on local interactions between cages. This approach works by approximating the geometry of the POCs to hard polyhedral shapes and condensing the intermolecular interactions to be approximated as the key interactions that drive the packing between different POC facets. In ref. 20, we used this coarse-graining approach to investigate how the directionality of the interactions between facets of hard octahedra affects the assembly of the octahedra in the solid state. With this relationship uncovered, we then compared the solid-state phase behaviour of the interacting hard octahedra to experimentally reported crystal structures of pseudo-octahedral POCs. This improved our understanding of the chemical functionality required to direct particular packing structures, elucidating design rules for targeted solid-state phase behaviour.

Here, instead of relating the phase behaviour spanned by a coarse-grained model to experimentally known structures, we test the ability of using local interactions determined by atomistic calculations on pairs of cages to predict the packing behaviour of POCs. This approach incorporates the dynamics of the rotational units of the molecule, as it optimises the structure of pairs of molecules, allowing them to relax and form favourable intermolecular interactions. With these atomistic calculations, the lowest energy configuration can inform the local interactions within our coarse-grained model which can be used to model the crystal structure of the cage. Unlike the desolvated pseudo octahedral POCs studied by us previously,^20^B11 has two different types of packing motifs along different axes, forming both columns of window-to-arene packing and sheets of window-to-window packing (Fig. 2(b and c)). This mixture of seemingly competing packing motifs makes B11 a useful test case for using dimer calculations to inform the coarse-grained model for predicting crystal structure formation *ab initio*.

Through this study, we show that local interactions are sufficient to drive the solid-state packing seen experimentally for B11. The workflow demonstrated here suggests that our coarse-grained methodology can be used to predict the packing of novel cages. Moreover, we demonstrate that chirality can be introduced into achiral cages through their solid-state interactions. Through the analysis of local interactions, we show that the achiral cage B11 favourably forms aggregates with unidirectional tilting of aromatic rings around a central benzene ring leading to a (*P*)- or (*M*)-propeller chirality. This analysis suggests that the formation of crystals can drive the realisation of chiral cages and that coarse-grained modelling can be used as a technique to design this chirality into materials. Although the resulting crystal structure formed from B11 is not homochiral, the analysis in this paper sheds light on a new route for introducing chirality into molecules through supramolecular interactions, which can be the first steps for realising homochiral crystal structures.

## 2 Results and discussion

### 2.1 Dimer calculations

**Fig. 3:**
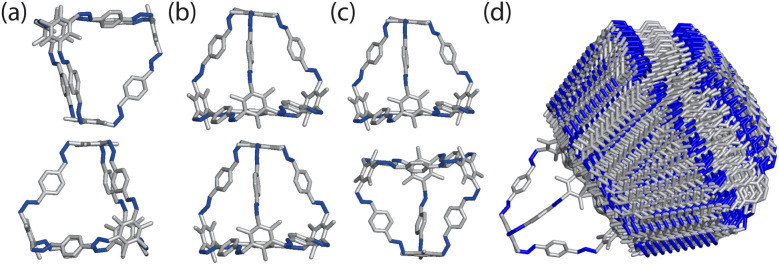
Packing motifs of the cages explored through dimer calculations. (a) Arene-to-arene, (b) window-to-arene, (c) window-to-window. (d) Overlay of all the dimers calculated for the window-to-window packing of B11.

To determine the local interactions between the cages, we used atomistic calculations to investigate the energetics of different packing motifs. We took a single molecule of the cage B11 from the experimentally reported structure (CCDC code PIFVAE)^19^ and geometry optimised it using the OPLS4 forcefield,^21^ which we have previously shown to perform well at reproducing cage conformations.^22^ With the optimised cage, we built dimers of B11 to examine the energetics of the most common packing motifs: window-to-window, window-to-arene, and arene-to-arene (Fig. 3). Each dimer was made by displacing a cage along the axis from the centroid of the cage to the centroid of the window or arene facet. To ensure we were exploring adequate configurational phase space for the dimers, we varied: (i) how far away each cage was from its dimer-pair, (ii) the angle of rotation of the cage around the displacement axis, and (iii) the cage's displacement perpendicular to the displacement axis. Details of the full configurational phase space explored are given in Section S1.†

For each configuration, the dimers were geometry optimised using OPLS4, constraining the atomic positions of the vertices of the cage to maintain the relative position/angles of the two cages. Dimers that were within 50 kJ mol^−1^ of the lowest energy configuration for their packing type were then optimised with no constraints. Unconstrained configurations that were within 30 kJ mol^−1^ of the lowest energy configuration for all dimer types were then further optimised using DFT. The cutoff of 30 kJ mol^−1^ was chosen as previous studies using DFT-D3 dimer calculations on imine cages showed that an energy difference with at least 20 kJ mol^−1^ between packing motifs were deterministic for their packing arrangement in the solid state.^23^ We increased this threshold to 30 kJ mol^−1^ to account for discrepancies between the force field and DFT calculations. For performing unconstrained dimer calculations on the results from the constrained dimer calculations, we chose a cutoff of 50 kJ mol^−1^ as studies using the OPLS3 force field for imine cages showed that a cutoff of 25 kJ mol^−1^ was sufficient for finding the lowest conformers using DFT calculations.^24^ This energy cutoff was doubled to ensure the lowest energy dimers were carried forward. The DFT calculations were performed with the mixed Gaussian and plane wave code CP2K/QUICKSTEP^25^ with the PBE functional,^26^ GTH-type pseudopotentials,^27^ molecular optimised TZVP-MOLOPT basis sets^28^ for all atoms and the Grimme-D3 dispersion correction.^29^ Details of the convergence criteria are given in the Section S1.†

**Fig. 4:**
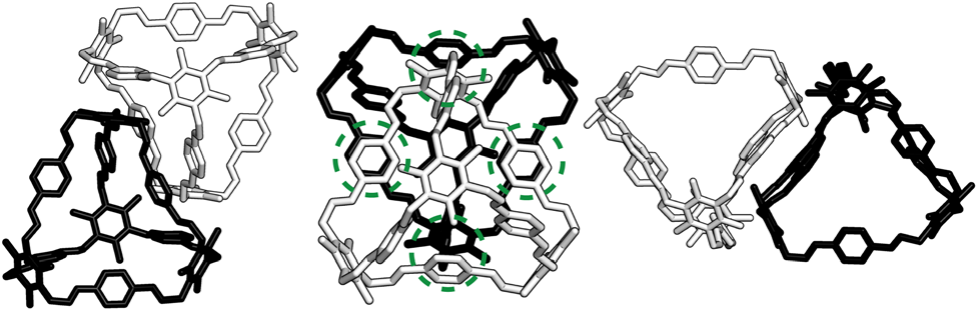
Different views of the lowest energy configuration of two interacting B11 cages. The cages are slipped off the central axis connecting the two centroids of the truncated tetrahedra to maximise π–π interactions between the aromatic rings along the edges of the cage and the arene facets, as circled in green.

From the constrained dimer calculations, the window-to-window dimers had the lowest energy structures, followed by the window-to-arene, and then the arene-to-arene (Table 1). This trend was also seen for the unconstrained dimer calculations. The energy difference between the constrained and unconstrained optimisation on the dimers shows that the window-to-window dimer is stabilised more than the other dimers (Table 1). This is because the unconstrained optimisation results in better π–π overlap between the cages, specifically between the arenes on the edges of one cage and vertices of the other. This π–π stabilisation does not occur, or at least not to the same extent, for the other dimers (Fig. S1†). The only dimers that were within 30 kJ mol^−1^ of the lowest energy configuration for the unconstrained dimer calculations, and thus taken forward for DFT calculations, had a window-to-window packing arrangement (Table 1). The DFT calculations showed that the lowest energy configuration of B11 is when the windows of the cages are anti-aligned, but slightly slipped off the centre of the facets (Fig. 4). In this configuration, the π–π interactions are maximised as the arenes on the vertices of the cages rotate to form π–π stacking with each other, as well as with the arene on the truncated facet of the tetrahedra. This leads to 4 π–π bonds between the two cages.

**Table 1:** Energies of the favoured dimers for each of the packing types from the dimer calculations as calculated using the OPLS4 forcefield. The energies are given relative to the lowest energy dimer

Packing type	Constrained optimisation (kJ mol^−1^)	Unconstrained optimisation (kJ mol^−1^)	Energy difference between constrained and unconstrained dimer (kJ mol^−1^)
Window-to-window	0	0	68.5
Window-to-arene	12.6	53.4	27.7
Arene-to-arene	56.2	109.9	14.8

### 2.2 Determining local interactions

At first look, the results from our dimer calculations are at odds with the crystal structure seen experimentally for B11, as B11's crystal structure has columns of window-to-arene packing that were calculated to be much higher in energy (>30 kJ mol^−1^) than window-to-window arrangements. However, due to the rotation of the arenes on the vertices to maximise π–π overlap, the packing at one of the windows of the cage affects the packing behaviour at the other windows beyond purely steric considerations.

**Fig. 5:**
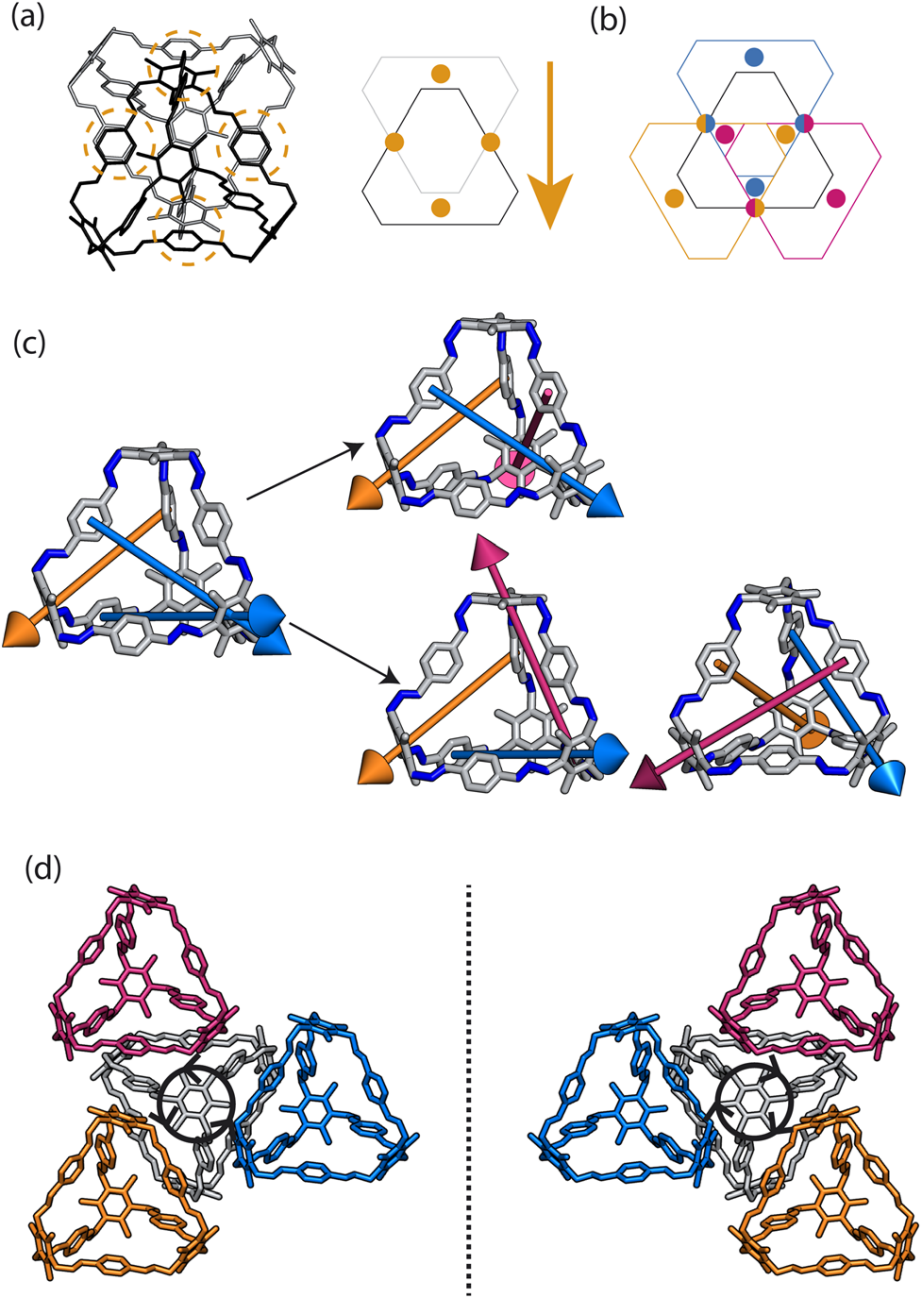
(a) Slipped arrangement to maximise π–π interactions where the cage in the foreground (black) is slipped relative to the cage in the background (grey). The slip direction is denoted as an arrow of the relative slip direction of the far cage to the close cage, and the π–π interactions are denoted as orange circles. (b) For a given window, shown in black, the neighbouring cage has a choice of three different slipped positions to adopt that are equivalent in energy, represented as the blue, orange, and pink positions of the cage relative to the black cage. (c) For a given initial slip direction of the first cage (orange), there are then two different arrangements the next cage can take around the central cage (blue arrows) to maximise the number of π–π interactions due to the rotation of the arenes on the vertices. For the third cage, there is then only one slip direction to maximise the π–π interactions (pink arrows). This leads to two arrangements of slip direction of cages which are mirror images of each other. (d) The two nearest neighbour arrangements of cages that maximise the number of nearest π–π interactions, related by a mirror plane, resulting in an anticlockwise (left) and clockwise (right) rotation of the arenes of the vertices about the axis perpendicular to the plane of the cages, as indicated by the black arrows.

For a given window, the neighbouring cage has a choice of three different positions in which to slip to adopt the lowest energy dimer configuration (Fig. 5(a and b)). The energy of each of these configurations is equivalent, but once one cage dimer is formed, its placement determines which positions neighbouring cages can sit at the other windows of the cage. This is because in order for there to be maximum π–π overlap, the arene rings in the vertices twist towards the neighbouring cage, which limits the orientations in which the arenes can form π–π interactions at neighbouring windows. Therefore, the choice of slip direction of one neighbouring cage influences the slip direction of the next. For a given slip direction, there are then two different arrangements the second cage can take around the central cage to maximise the number of slipped window-to-window interactions, indicated by the blue arrows in Fig. 5(c). When the slip direction of the second cage is chosen, there is only one window and slip direction accessible for a third cage to take to form 4 π–π interactions, represented as the pink arrows in Fig. 5(c). There is then no way to place another cage by the final window facet that would result in the same number of π–π interactions and as such the maximum number of cages in a slipped window-to-window fashion around a cage is three. As the initial choice of slip direction of the first cage is equivalent by rotation, the choice of placement by the second cage leads to two different arrangements of neighbouring cages around each cage that are mirror images of each other (Fig. 5(d)).

The combined effect on the rotation of the arenes in the vertices leads to propeller-like behaviour of three of the arenes in the central cage around the axis perpendicular to the one containing the neighbouring cages. The two different arrangements of cages leads to the different possible isomers of this propeller-like rotation: clockwise and anticlockwise. Therefore, the dimer calculations suggest that the local supramolecular interactions between the cage could incite chiral behaviour in the cages. But are these local interactions enough to drive the solid-state phase behaviour seen experimentally?

The “knock-on effect” due to the twisting of arenes at different facets limits the local interactions that are available to each cage, which means window-to-window packing is not necessarily favourable at all window facets, as the π–π interactions can only be maximised at three of the four window facets. As these interactions only lead to packing at three of the four windows, we need to consider what other interactions can occur at the last facet in order to predict the packing behaviour of the cages. From dimer calculation results, the next lowest energy packing motif is window-to-arene (Table 1). The combination of the three slipped window-to-window packing and the competing window-to-arene interactions may be the cause of the planes of window-to-window packing and window-to-arene columns seen in the experimental structure.

### 2.3 Hard particle Monte Carlo simulations

**Fig. 6:**
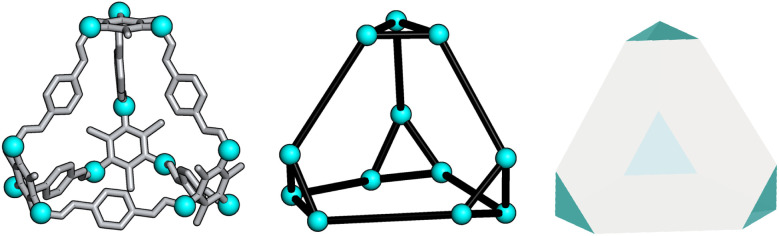
Relation of B11 to a hard truncated tetrahedra where the vertices of the cage are shown as cyan spheres.

To determine whether the analysis of the local interactions is enough to produce the crystalline phase behaviour seen experimentally for B11, we ran hard-particle Monte Carlo (HPMC) simulations to examine the effect of these local interactions on the phase behaviour of the material. The Monte Carlo simulations were performed using HPMC,^30^ a plugin to the HOOMD-blue simulation toolkit.^31^ These simulations considered the particles as hard truncated tetrahedra. The truncation of the tetrahedron is given by a truncation parameter *t* as defined in ref. 32, where the truncated tetrahedron has four equilateral triangles with edge length *σ*(*t*/2) and four hexagons with two alternating edge lengths of *σ*(*t*/2) and *σ*(1 − *t*). For our simulations we set *σ* = 1 for simplicity and we set the truncation parameter by calculating the ratio of the lengths of the different vertices in B11, resulting in *t* = 0.5 (Fig. 6). This shape is also known as a space-filling truncated tetrahedron.^32^

The HPMC simulations perform moves that rotate or translate the truncated tetrahedra. If the move results in an overlap of the hard particles, it is rejected, whereas when there is no overlap between the particles the move can be accepted. As with our previous work,^20^ we set up additional acceptance criteria if there is no overlap by adding an interaction potential between the hard particles.

#### 2.3.1 Patchy particle potential

To determine the effect of the local intermolecular interactions on the crystalline phase behaviour of the POC B11, we implemented HPMC simulations on hard truncated tetrahedra with directional interactions between the facets. Here the hard truncated tetrahedra were decorated with sticky patches mimicking the intermolecular interactions through the potential *V*_ij_. This potential takes the same form as the interactions laid out in ref. 20, where there are three components; *V*_LJ_, *V*_ang_, and *V*_tor_ which correspond to a Lennard-Jones potential, angular, and torsional modulation term respectively:^33,34^1
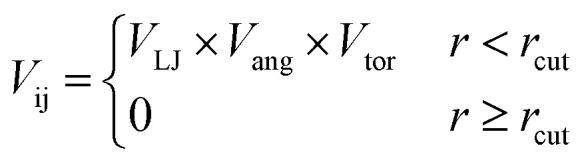
2
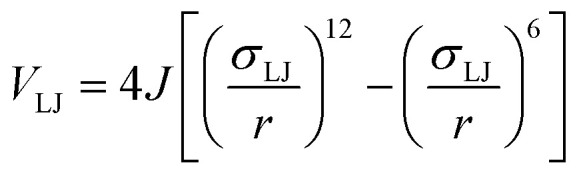
3
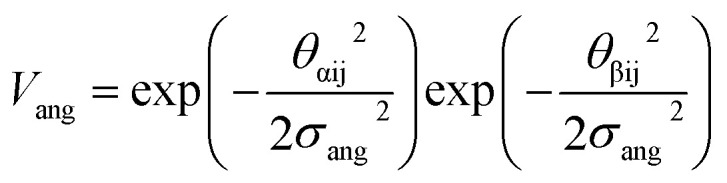
4
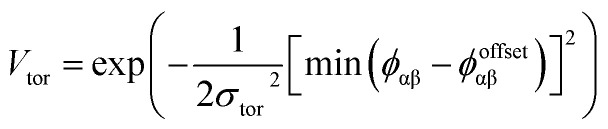


The patches on particles i and j are labelled α and β respectively. A 2D representation of the patchy particle is shown in Fig. S2.† For the simulations here, *σ*_LJ_, normally the measure of the diameter of the particles, is set to 0.75 Å for the patches leading to slipped window-to-window configurations, and 0.95 Å for window-to-arene interactions. For the patches between the window-to-arene packing, this value is slightly larger than the minimum distance between the hard truncated tetrahedra. This is to allow for a small gap between the hard particles, which would exist between neighbouring cages due to their van der Waals radii. For the patches leading to window-to-window packing, this value is only marginally larger than their closest point 
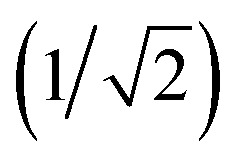
 as the cages have to be very close in order to have overlap between the aromatic rings on the truncated facets and vertices. *r* is a measure of the distance between the centroids of the neighbouring particles, and the cutoff distance, *r*_cut_, is set to 1.92*σ*_LJ_ for the smallest *σ*_LJ_, *i.e.* the window-to-window interaction, so that the patches interact only with nearest neighbour polyhedra. *J* is the measure of the interaction strength between the hard truncated tetrahedra.

The angular term, *V*_ang_, is a measure of the directionality of the interactions between patches on adjacent particles. Here, *θ*_αij_ is the angle between the patch vector and the vector between the two neighbouring particles, *r*_ij_, and *σ*_ang_ determines the severity of the energetic penalty for particles deviating from perfect alignment. The form of this interaction potential is such that a larger value of *σ*_ang_ allows for worse alignment of the centroids of the patches. In this work, as the interactions between the cages is dictated by π–π interactions that are inherently directional, we set *σ*_ang_ = 0.3. This value was chosen based on our previous study of using patchy particle potentials to study the phase behaviour of hard octahedra. In that study, instances where *σ*_ang_ = 0.1 sometimes failed to form a cluster, but for a given interaction type, the simulations showed consistent solid-state phase behaviour when *σ*_ang_ ranged from 0.2 to 0.4.^20^

The final term is the torsional term, *V*_tor_, which describes the modulation of the potential with rotation of the particle about the interparticle vector *r*_ij_. Here, *ϕ*^offset^_αβ_ is the preferred torsional angle between patches α and β, whereas *ϕ*_αβ_ is the actual torsional angle. The Gaussian function is set out such that there is an energy minimum where the two angles are the same. This term was used to set the relative alignment of the cages, determining if the orientation of neighbouring cages is the same or different, where the preferred torsional angles in the simulations were based on the results from the dimer calculations. Here, the window-to-window dimers have cages with different orientations which are anti-aligned (Fig. 4) such that interactions leading to window-to-window packing have *ϕ*^offset^_αβ_ was set to π/3. For the window-to-arene packing, there were multiple low energy motifs for different orientations between the dimers and thus there was no torsional preference and therefore for the interactions resulting in window-to-arene packing, *V*_tor_ was set to 1. Similarly to *σ*_ang_, *σ*_tor_ dictates the energetic penalty of the particles deviation from the perfect torsional angle. For the window-to-window packing, as in ref. 20, we set *σ*_tor_ = 2*σ*_ang_ in the simulations.

**Fig. 7:**
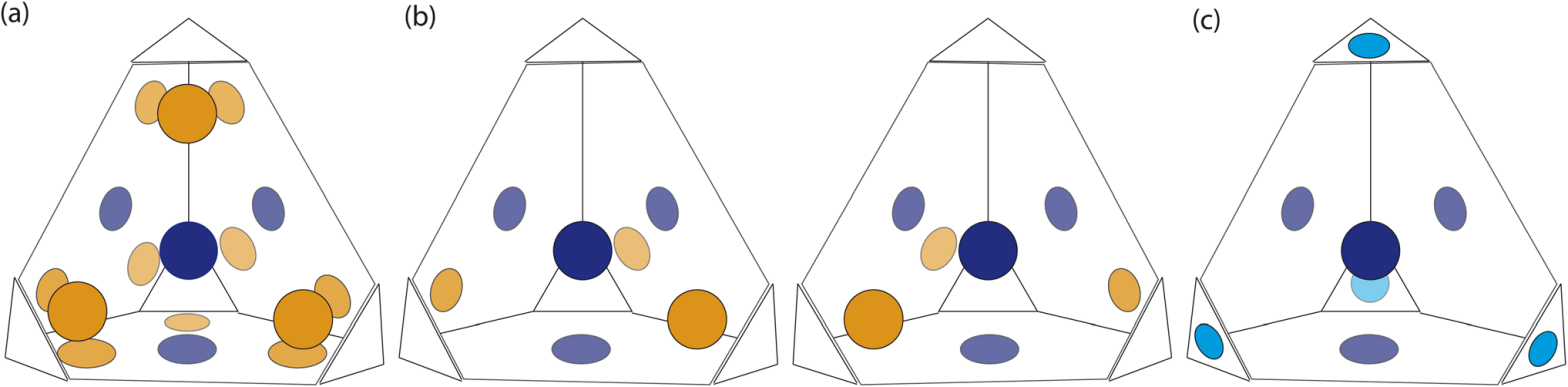
Schematic hard truncated tetrahedra with attractive interactions between blue patches and orange patches. Each window has one blue patch and three orange patches which are off-centred towards the arene facets. Interactions between the orange and blue patches represent the slipped window-to-window configurations that occur between the cages due to the interactions between the arenes on the vertices and facets. (a) All possible interactions based on the dimer calculations, not taking into account the influence of cages interacting at neighbouring facets. (b) Taking neighbouring facets into account, there are two types of hard truncated tetrahedra with different patches that are mirror images to each other. (c) Interactions between cyan and blue patches on neighbouring polyhedra that lead to window-to-arene packing.

Based on the dimer calculations, the interactions between cages lead to off-centre window-to-window packing between the cages to maximise π–π interactions. Excluding any next-nearest neighbour effects from the arenes twisting as described in Section. 2.2, the favourable positions of neighbouring cages to maximise π–π interactions is equivalent to attractive interactions between the orange patches, taking into account the slip direction between the cages, and blue patches on the centre of the window facets, of neighbouring truncated tetrahedra as shown in Fig. 7(a). To include the constraints on the slip directions necessary to maximise π–π overlap due to interactions at neighbouring facets, the number of patches available were decreased. The two types of arrangements around each cage, as shown in Fig. 5(b and c), similarly produce two different arrangements of patches on the hard truncated tetrahedra that are mirror images of one another (Fig. 7(b)). As these local interactions would lead to one window remaining vacant, we added a secondary interaction based on the next most favourable packing motif as determined from the dimer calculations; a window-to-arene interaction. This was implemented by adding attractive interactions between the blue and cyan patches shown in Fig. 7(c), which represent the centroids of the window and arene facets respectively.

#### 2.3.2 Simulation details

The simulations were performed in the NVT ensemble where the temperature was slowly cooled from *k*_B_*T* = 1.12125 over the transition point *k*_B_*T* ≈ 1 with temperature steps of *T*_*i*+1_ = *T*_*i*_ × 0.975. The simulations were run for 8 hours using 64 cores on Imperial College London's Research Computing Service facilities which resulted in 9 temperatures being sampled. For the first 8 temperatures, 10^8^ timesteps were sampled, where a random number of particles are moved in the cell for each timestep. As the time for each timestep to be completed depends on the number of interactions being calculated, once the polyhedra form a cluster, the time taken to complete each timestep increases. Due to the finite wall time of the simulations, the simulations were terminated before finishing all time steps. The cluster outputted on termination is considered to be representative of the phase behaviour at all lower temperatures.

Our HPMC simulation contained 512 particles in cubic boxes with a box length of 16 Å. The simulations were performed at very low density to ensure the clusters formed had no mechanical stress or structural defects. Within our simulations, we had a 50 : 50 mixture of two types of truncated tetrahedral particles which had the two different “chirality” of patches (Fig. 7(b)). The interactions between the cages were not biased, such that it would be equally likely for cages of the same or different chirality to interact. Therefore if the cages preferably form a homochiral crystal, we would expect the simulations to form two separate clusters with only one isomer of the cage. Alternatively, if interactions between different chirality interactions are preferable, only one cluster would form with a racemic mixture of the isomers.

As there were two types of patchy interactions leading to attractive interactions between windows, and windows and arenes, there are two interaction strengths to consider for *J* in eqn (3). These are referred to as *J*_ww_ and *J*_wa_ for the interaction between the orange and blue patches, and cyan and blue patches as shown in Fig. 7. As the absolute energy scale is not important to the results of this study, for simplicity the interaction strengths were chosen for the simulation such that the transition temperature *T*_t_ in which a cluster forms occurs when *k*_B_*T*_t_ ≈ 1 for the dominant interaction between the windows, which occurred when *J*_ww_ = 60. *J*_wa_ was chosen to be smaller than *J*_ww_, such that without the interactions that favoured window-to-window packing, the cluster would form with window-to-arene packing as the dominant motif at *k*_B_*T*_t_ ≈ 0.75, resulting in a value of *J*_wa_ = 15. This follows the dimer calculation results, which showed that the interactions that lead to window-to-window packing are preferred.

### 2.4 Crystalline phase behaviour

**Fig. 8:**
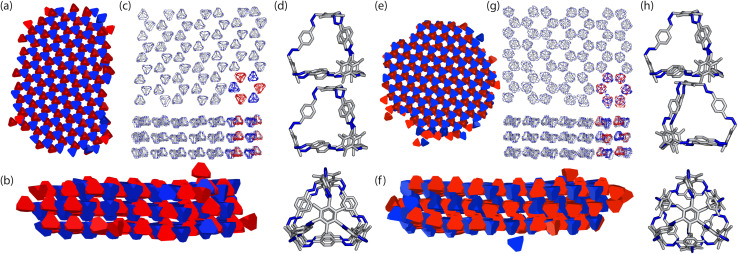
(a, b, e and f) Results of the two different crystal structures to form from the HPMC simulations where the truncated tetrahedra with the two different interactions are coloured in red and blue to represent their different chiralities. (c and g) The corresponding structure with the cage B11 where a subsection of cages coloured to show the corresponding chirality of the cages. (d and h) The different window-to-arene packing between the two crystal structures where the cages are aligned and anti-aligned respectively.

To ensure consistency, we ran the simulations five times which lead to the formation of two different crystal structures, as shown in Fig. 8. The formation of the two distinct structures from our stochastic simulations suggests that both phases are low-energy states, potentially representing degenerate states of the coarse-grained model. These clusters were formed of a racemic mixture of the truncated tetrahedra with different chirality interactions. Both clusters had layers of cages where each cage was surrounded by three cages with interactions of opposing chirality. As the interactions between the particles were unbiased, such that it was equally likely to form a homochiral or racemic crystal structure, this result suggests that to maximise the π–π overlap between neighbouring cages, the interactions occur between cages of opposite chirality, which is in agreement with the experimentally reported structure.^19^ Perpendicular to the planes, the cages form columns that are packed in a window-to-arene fashion. The two clusters differ in the orientation of the cages between the layers. Due to the equivalent energy of the window-to-arene dimers for different alignment between the cages, our interactions between the windows and arenes had no preference in orientation. This led to the formation of two different crystal structures which maximise the window-to-arene packing between the layers, where the cages between the layers were either aligned or anti-aligned, such the chirality of the cages switched between the layers.

With these two clusters, using a similar approach to our previous work,^20^ we determined the space groups of the corresponding crystals structures. Details of this process can be found in Section S3.1.† For the cluster where the cages were aligned between the layers, the space group was found to be *P*3̄, matching the experimental structure of B11 reported in the literature (CCDC code PIFVAE^19^). For the cluster where the cages were anti-aligned between the layers, the space group was *P*3̄*c*1, which has not been reported experimentally. With these two structures, we sought to determine which one was more energetically stable.

As our crystal structures solved from the coarse-grained model were at unrealistic densities (≈0.6 g cm^−3^) we used the molecule to crystal function in CrystalMaker® with the DFT optimised molecule, to create the two crystal structures. The crystal structures were created such that the density was maximised without any overlap between the cage molecules within the crystal structure, leading to crystal structures with the lattice parameters *a* = 20.5 Å, *c* = 14.1 Å and a density of 0.9176 g cm^−3^ for *P*3̄ and *a* = 20.5 Å, *c* = 28 Å and a density of 0.9241 g cm^−3^ for *P*3̄*c*1.

**Fig. 9:**
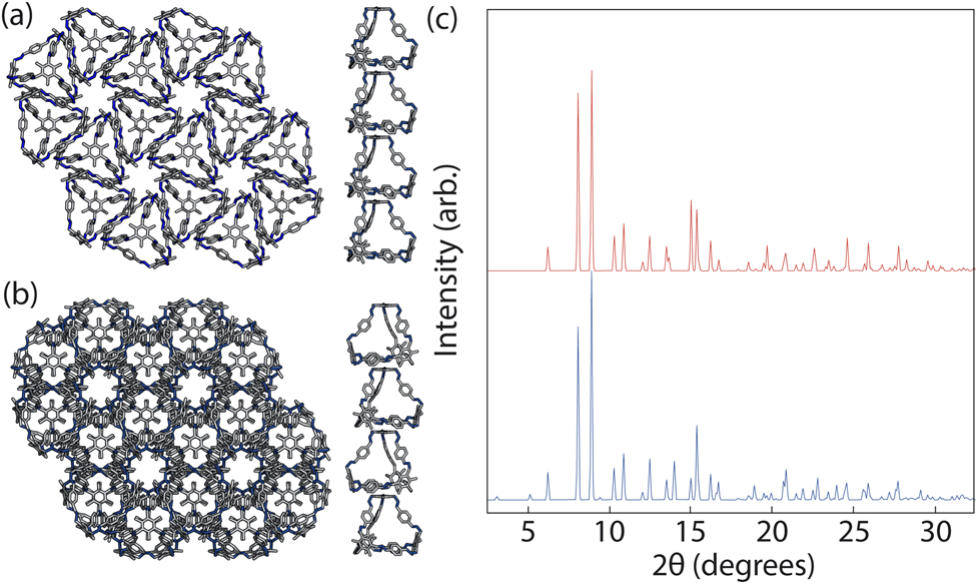
DFT optimised crystal structures of (a) the predicted *P*3̄ structure where the cages are aligned between the layers, and (b) the predicted *P*3̄*c*1 structure where the cages are anti-aligned between the layers. (c) The PXRD patterns of the two optimised structures where the red and the blue patterns are the calculated patterns for the *P*3̄ and *P*3̄*c*1 crystal structure respectively.

With these structures, we performed full geometry optimisation using DFT calculations, producing the two structures shown in Fig. 9(a and b), details of which are in Section. S3.2.† We then compared the similarity of the fully optimised *P*3̄ crystal structure to the experimentally reported structure using COMPACK.^35^ For an overlay of 15 molecules excluding hydrogens, a RMSD = 0.234 Å was observed (Fig. S4†). To determine which one was more energetically stable, we compared the energies of the two structures and found the *P*3̄*c*1 structure was 3.2 kJ mol^−1^ higher in energy than *P*3̄. This energy difference is on the order of error for the DFT calculations and thus considered negligible, evidencing that our coarse-grained model that only considered nearest neighbour interactions was able to accurately encapsulate the thermodynamic phase behaviour of B11. Whilst more accurate DFT approximations could be used to further determine the energy differences between the structures, DFT-D3 calculations are considered state-of-the-art and are commonly used to evaluate the energy difference between different polymorphs^36^

Although these calculations show that both structures may be equally thermodynamically stable, only one crystal structure has been reported experimentally. Solvent is known to have an effect on the packing behaviour of POCs,^37^ and B11 itself has been reported to form two polymorphs based on solvation.^19^ As our dimer calculations were on desolvated cages, we only expect our simulations to produce the experimentally reported desolvated structure, which was reported to contain disordered water molecules within the pores. The presence of the water molecules could stabilise *P*3̄ relative to *P*3̄*c*1, driving the crystalline phase behaviour. To test this theory we optimised each of the structures with one water molecule in one of three partially occupied sites within each cage and compared the energies of the *P*3̄*c*1 and *P*3̄ phases. These results show that the *P*3̄*c*1 structure is higher in energy by 4.1 kJ mol^−1^. Although this increase in energy difference could be responsible for the observation of only one phase experimentally, we also suggest that perhaps as the PXRD patterns of the two structures are very similar (Fig. 9(c)), containing many of the same reflections that only differ in intensity, the two structures may coexist in the bulk material where the relative alignment between layers of the cages is statistically distributed. This result is also seen in our simulations as for simulations with a larger value of *J*_wa_, where *J*_wa_ = 25, the clusters contained more than three layers which resulted in a statistical distribution of the relative orientation of the cages between the layers (aligned or anti-aligned).

## 3 Conclusions

We have shown that using a combination of dimer calculations and coarse-grained models through hard particle Monte Carlo simulations with patchy interactions, we can recreate the packing behaviour seen for the POC B11. Our dimer calculations allowed us to analyse the local interactions between POCs to inform coarse-grained models and use them to predict the cage's solid-state phase behaviour. This analysis shows that the local interactions in B11 cause rotations of the arenes on the vertices, which drives the solid-state phase behaviour of the cage and results in propeller-like orientations of the aromatic rings on the vertices of the cages. Our simulations therefore demonstrate how supramolecular interactions in the solid-state can drive chirality in cages on a molecular level without the need for a chiral guest.

Although these supramolecular interactions produce chirality within the cage, our results are in agreement with experiments, showing that a racemic mixture of the propeller orientations form on crystallisation instead of crystallising into a homochiral crystal structure. However, chiral interactions may be able to be designed into cage structures such that we can target a cage which forms an enantiopure crystal instead of racemic. For example, by placing hydrogen bonding groups on the vertices where the hydrogen bonding network results in preferential isomers in the crystal structure. The results from this paper are therefore a first step toward creating design strategies for realising chiral phenomena in cages such as circularly polarized luminescence for spintronics. By varying the molecular shape and the types of chiral interactions abstractly through coarse-grained models, we could help to inform design principles for targeted solid-state phase behaviour.

Aside from supramolecular chirality, successfully determining the local interactions based on dimer calculations and using them to inform the coarse-grained model has interesting consequences for using this methodology to predict the packing behaviour of novel POCs. Our results highlight how these simulations can provide insights into disorder within the crystal structure. For example, for some of our simulations we get a mixture of layers of cage orientations, suggesting that these cages form in a statistical distribution on crystallisation instead of a completely ordered structure. Moreover, unlike many traditional techniques such as crystal structure prediction, our methodology inherently takes into account the rotational flexibility of the structures due to the geometry optimisation of the dimers. Although not necessary here, for other molecules where conformational flexibility leads to many low energy conformers, using a similar approach we could do a full conformer search and perform dimer calculations on combinations of each of the low energy conformers to find the lowest energy local interactions. This could help mitigate a long term problem in crystal structure prediction, where an increase in the number of conformers leads to a large increase in the computational expense to ensure thorough exploration of the conformational landscape. The dimer calculations presented here are relatively computationally inexpensive, and increasing the number of types of components in the HPMC simulations in order to include multiple conformers does not increase the computational expense of the simulations. Therefore, the methodology laid out in the paper could provide a computationally inexpensive route to predicting polymorphic behaviour of flexible molecules orders of magnitude faster than current computational techniques.

## Author contributions

E. H. W. conceptualised and designed the project, carried out and analysed the simulations. K. E. J. supervised the project and acquired funding. E. H. W. wrote the manuscript and all authors contributed to the final version.

## Conflicts of interest

There are no conflicts to declare.

## Supplementary Material

SC-015-D4SC04430D-s001

SC-015-D4SC04430D-s002

SC-015-D4SC04430D-s003

SC-015-D4SC04430D-s004

SC-015-D4SC04430D-s005

SC-015-D4SC04430D-s006

## Data Availability

The software used to run the HPMC simulations is a plugin in the HOOMD-blue simulation toolkit which can be found at https://github.com/glotzerlab/hoomd-blue. The version of the code employed for this study is 2.9.7. An example script of how HOOMD-blue is used to predict the packing of porous organic cages can be found at https://github.com/ewolpert1/CG_cages. Example clusters of the two different crystal structures produced in the HPMC simulations and their corresponding DFT optimised cifs are included in the ESI.†
