# Supplementary material for: Introducing chirality in porous organic cages through solid-state interactions
Source: Chem Sci. 2024 Sep 18;15(40):16519–28. doi: 10.1039/d4sc04430d (PMC11420649; doi:10.1039/d4sc04430d)
Supplement: SC-015-D4SC04430D-s005 [file SC-015-D4SC04430D-s005.pdf]

Supplementary Information (SI) for Chemical Science.
This journal is © The Royal Society of Chemistry 2024

Supporting Information: Introducing chirality in porous organic cages through solid-state interactions

Emma H. Wolpert* and Kim E. Jelfs*

Department of Chemistry, Imperial College London, Molecular Sciences Research Hub, White City Campus, Wood Lane, London, W12 0BZ, UK

E-mail: e.wolpert@imperial.ac.uk,k.jelfs@imperial.ac.uk

Contents

1 Details of dimer calculations **S-3**

1.1 Configurational phase space exploration . . . S-3

1.2 Dimer configurations . . . S-4

2 Patchy particle model **S-5**

3 Crystal structure determination **S-6**

3.1 Structural determination . . . S-6

3.2 DFT details . . . S-7

3.3 Experimental structure and calculated structure overlay . . . S-8

References **S-9**

1 Details of dimer calculations

1.1 Configurational phase space exploration

To calculate the dominant packing motifs between the cages, we looked at the interactions between the arene-to-arene, window-to-arene, and window-to-window facets (Fig. **??**(a-c)). To ensure we were exploring adequate configurational phase space for the dimers, we varied: (i) how far away each cage was from its dimer-pair in increments of 1 Å between 10 and 19 Å for window-to-window and window-to-arene and between 15 and 19 Å for arene-to-arene due to the larger distance to the facet, (ii) the angle of rotation of the cage around the displacement axis (0,30,60,90), and (iii) its displacement perpendicular to the displacement axis, defined by a sphere where θ and ϕ were varied between and $\frac{4}{20}\pi$ and $\frac{9}{20}\pi$, and $\frac{3}{20}\pi$ and $\frac{8}{20}\pi$ at increments of $\frac{1}{20}$ respectively (Fig. **??**). For each configuration, the dimers were geometry optimised using OPLS4,[S1] constraining the atomic positions of the vertices of the cage to maintain the relative position/angles of the two cages. Dimers that were within 50 kJ mol^{-1} of the lowest energy configuration for their packing type were then full geometry optimised with no constraints. Unconstrained configurations that were within 30 kJ mol^{-1} of the lowest energy configuration were then further optimised using DFT using the mixed Gaussian and plane wave codes CP2K/QUICKSTEP[S2] with the PBE functional,[S3] GTH-type pseudopotentials,[S4] molecular optimised TZVP-MOLOPT basis sets[S5] for all atoms and the Grimme-D3 dispersion correction.[S6] The cutoff for the plane wave grid used in these calculations was 400 Ry with a relative cutoff of 60 Ry.

1.2 Dimer configurations

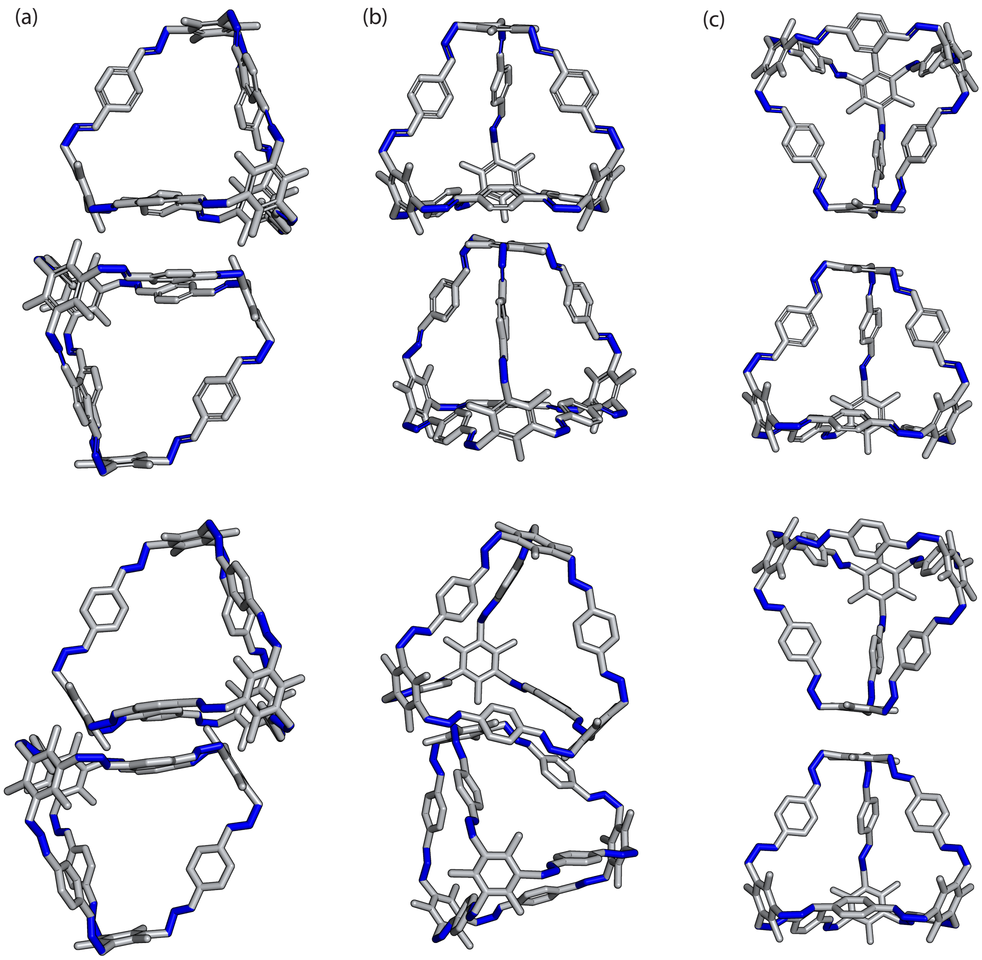

Figure S1: (a) Window-to-window, (b) window-to-arene, and (c) arene-to-arene dimers of the lowest energy unconstrained optimisation optimisations after constrained optimisation (top) and after unconstrained optimisation (bottom).

2 Patchy particle model

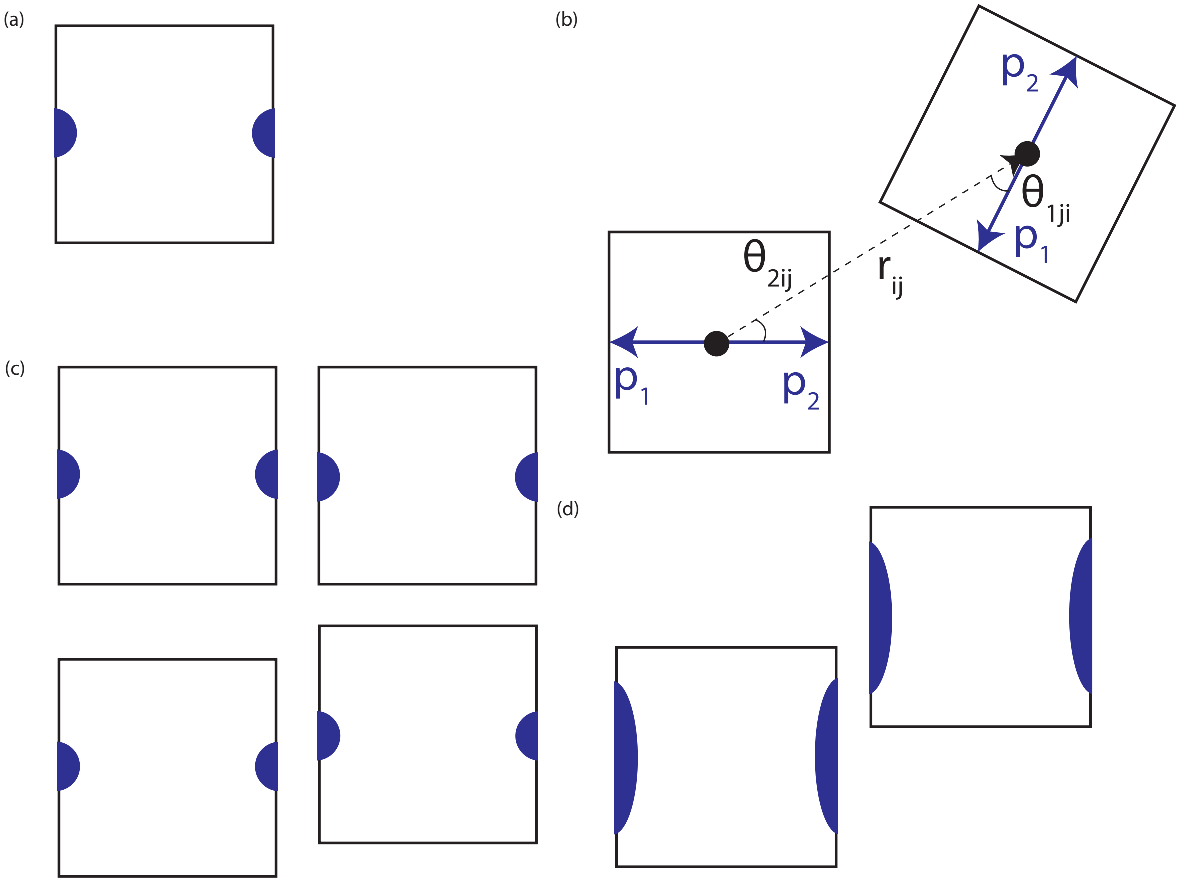

Figure S2: A 2D example of the patchy particle model. (a) A patchy particle with two attractive patches shown in blue. (b) Representation of the terms calculated for the patchy particle model. Here there are two particles: i and j which each have two patches p_1 and p_2. The patches closest to the interparticle vector, r_{ij}, interact which in this case is p_2 on particle i and p_1 on particle j. (c) The lowest energy configuration for the patchy particle is shown at the top, with the maximum likely displacement between two particles with a small patch width (small σ_{ang}) shown at the bottom. (d) Maximum likely displacement between two particles with a large patch width (large σ_{ang}).

3 Crystal structure determination

3.1 Structural determination

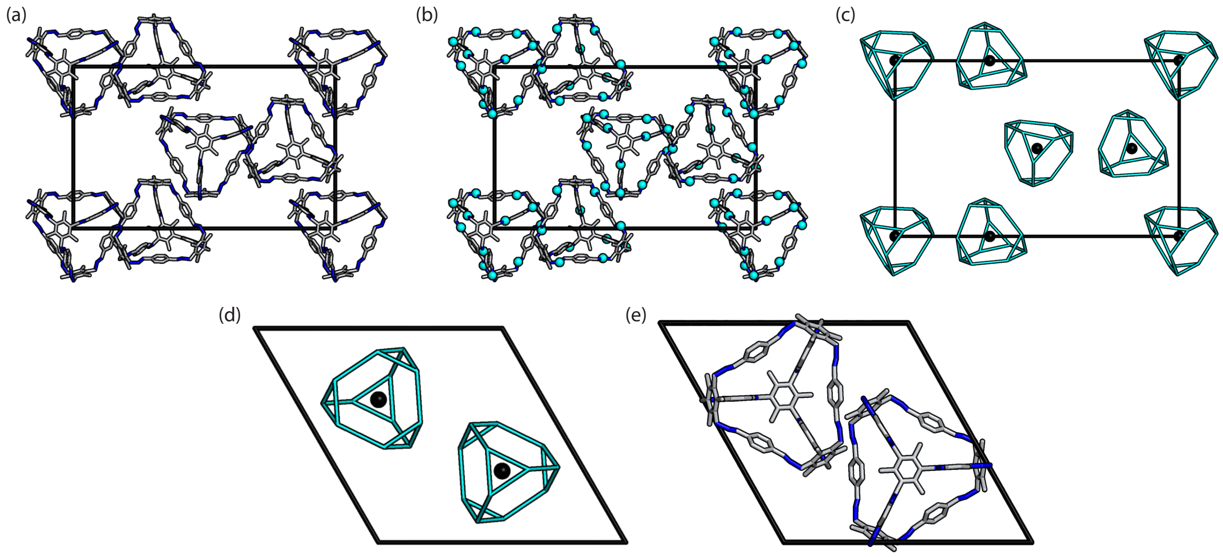

Figure S3: Overview of the structural determination process. (a) Unit cell (shown in black) abstracted from a cluster from the simulations. (b) The nitrogen atoms from the vertices (cyan) which were used to retain the symmetry of the molecule but reduce the degrees of freedom. (c) Coarse-grained structure with just the central position of the cages (black spheres) and nitrogen atoms used in $FINDSYM$ to get the space group of the structure. (d) Outputted structure from $FINDSYM$ with the space group $P\overline{3}$. Crystal structure obtained by converting the unit cell in (a) to the symmetry resolved unit cell, applying the symmetry operations of $P\overline{3}$ and merging the atoms that sit on the same crystallographic sites. The space between the representative cages was larger for the coarse-grained structure in order to find the space group easier.

Using custom made code, we took clusters of the truncated tetrahedra formed during the simulations, transformed them onto clusters of the cage **B11** and identified a representative unit cell of the cages (Fig. S3(a)). During this process the centre of each cage was "neatened" in order to put them on likely high symmetry sites *i.e.* $\frac{1}{4}, \frac{2}{4}, \frac{3}{4}$ or $\frac{1}{3}, \frac{2}{3}$ *etc.* As the orientation of the cages within the unit cell were slightly disordered due to the nature of the simulations, we created a coarse-grained representation of each cage in order to use traditional symmetry finding algorithms. This coarse-graining process involved converting each cage into an atom representing the centre of the molecule, as well as the nitrogen atoms at the vertex of each

of the cages to preserve the symmetry of cage (Fig. S3(b-c)). Using this structure, we employed *FINDSYM*[S7,S8] to determine the space group (Fig. S3(d)). We then applied the transformation invoked by *FINDSYM* to the unit cell containing the cages, applied the symmetry conditions, and merged the atomic positions which were at the same sites to get the ordered cage structure with a fully solved space group (Fig. S3(e)). For the $P\overline{3}c1$ cluster, we used *FINDSYM* twice as initially the symmetry was found to be $P321$, which when we uploaded the structure to *FINDSYM* again, the symmetry increased further to $P\overline{3}c1$.

3.2 DFT details

The crystal structures for the cage in the space group $P\overline{3}$ and $P\overline{3}c1$ were fully optimised using DFT calculations. The DFT calculations used the mixed Gaussian and plane wave codes CP2K/QUICKSTEP[S2] with the PBE functional,[S3] GTH-type pseudopotentials,[S4] molecular optimised TZVP-MOLOPT basis sets[S5] for all atoms and the Grimme-D3 dispersion correction.[S6] The cutoff for the plane wave grid used in these calculations was 1000 Ry with a relative cutoff of 60 Ry.

3.3 Experimental structure and calculated structure overlay

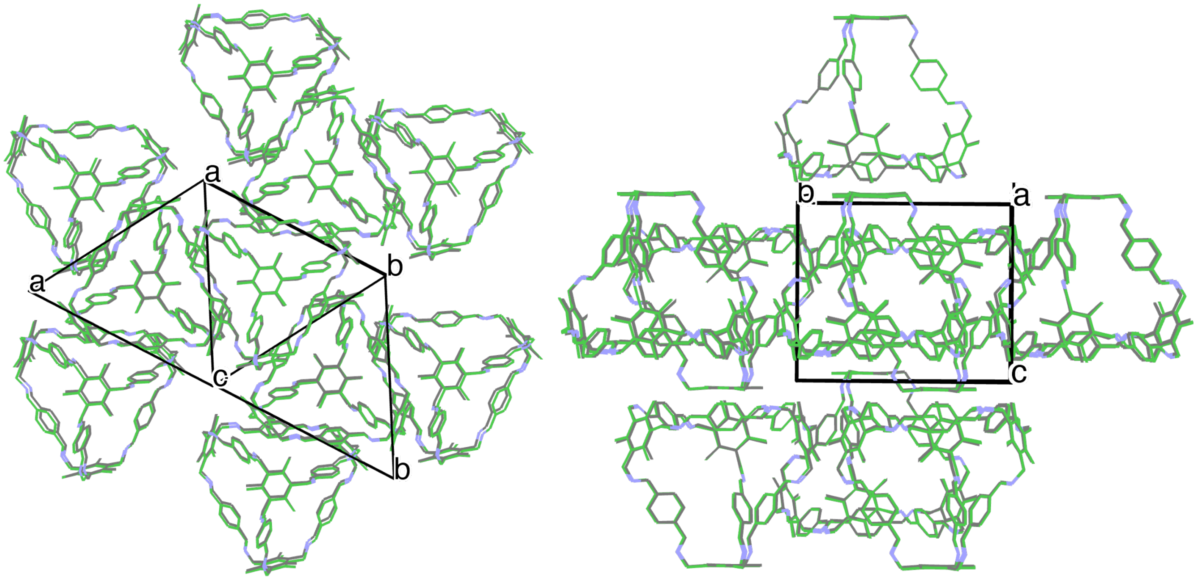

Figure S4: Molecular overlay of the **B11** single crystal X-ray structure (CSD reference code: PIFVAE) and the fully optimised $P\bar{3}$ crystal structure produced from the coarse-grained simulations. For an overlay of 15 molecules excluding hydrogens, a RMSD = 0.234 Å was observed. The experimental (predicted) structure is shown in grey (green).

References

(S1) Lu, C.; Wu, C.; Ghoreishi, D.; Chen, W.; Wang, L.; Damm, W.; Ross, G. A.; Dahlgren, M. K.; Russell, E.; Von Bargen, C. D.; Abel, R.; Friesner, R. A.; Harder, E. D. OPLS4: Improving Force Field Accuracy on Challenging Regimes of Chemical Space. *Journal of Chemical Theory and Computation* **2021**, *17*, 4291–4300, Publisher: American Chemical Society.

(S2) Kühne, T. D.; Iannuzzi, M.; Del Ben, M.; Rybkin, V. V.; Seewald, P.; Stein, F.; Laino, T.; Khaliullin, R. Z.; Schütt, O.; Schiffmann, F.; Golze, D.; Wilhelm, J.; Chulkov, S.; Bani-Hashemian, M. H.; Weber, V.; Borštnik, U.; Taillefumier, M.; Jakobovits, A. S.; Lazzaro, A.; Pabst, H.; Müller, T.; Schade, R.; Guidon, M.; Andermatt, S.; Holmberg, N.; Schenter, G. K.; Hehn, A.; Bussy, A.; Belleflamme, F.; Tabacchi, G.; Glöß, A.; Lass, M.; Bethune, I.; Mundy, C. J.; Plessl, C.; Watkins, M.; VandeVondele, J.; Krack, M.; Hutter, J. CP2K: An electronic structure and molecular dynamics software package - Quickstep: Efficient and accurate electronic structure calculations. *The Journal of Chemical Physics* **2020**, *152*, 194103.

(S3) Perdew, J. P.; Burke, K.; Ernzerhof, M. Generalized Gradient Approximation Made Simple. *Physical Review Letters* **1996**, *77*, 3865–3868, Publisher: American Physical Society.

(S4) Goedecker, S.; Teter, M.; Hutter, J. Separable dual-space Gaussian pseudopotentials. *Physical Review B* **1996**, *54*, 1703–1710, Publisher: American Physical Society.

(S5) VandeVondele, J.; Hutter, J. Gaussian basis sets for accurate calculations on molecular systems in gas and condensed phases. *The Journal of Chemical Physics* **2007**, *127*, 114105.

(S6) Grimme, S.; Antony, J.; Ehrlich, S.; Krieg, H. A consistent and accurate ab initio

parametrization of density functional dispersion correction (DFT-D) for the 94 elements H-Pu. *The Journal of Chemical Physics* **2010**, *132*, 154104.

(S7) Stokes, H. T.; Hatch, D. M. *FINDSYM* : program for identifying the space-group symmetry of a crystal. *Journal of Applied Crystallography* **2005**, *38*, 237–238.

(S8) Stokes, H. T.; Hatch, D. M.; Campbell, B. J. FINDSYM, ISOTROPY Software Suite, iso.byu.edu. `iso.byu.edu`.
